# Ceramic versus Composite Resin Polishing Systems on the Surface Roughness of Milled Leucite-Reinforced Ceramics

**DOI:** 10.3390/medicina59061048

**Published:** 2023-05-29

**Authors:** Carlos A. Jurado, Saad Alresayes, Silvia Rojas-Rueda, Ali Alqahtani, Akimasa Tsujimoto, Nicholas G. Fischer, Kelvin I. Afrashtehfar

**Affiliations:** 1 Department of Prosthodontics, University of Iowa College of Dentistry and Dental Clinics, Iowa City, IA 52242, USA; carlos-jurado@uiowa.edu; 2 Department of Prosthetic Dental Sciences, King Saud University College of Dentistry, Riyadh 11545, Saudi Arabia; salresayes@ksu.edu.sa; 3 School of Dentistry, Pontifical Xaverian University, Bogota 110231, Colombia; rojassmarcela@javeriana.edu; 4 Department of Periodontics and Community Dentistry, King Khalid University College of Dentistry, Abha 62529, Saudi Arabia; aaljabbar@kku.edu.sa; 5 Department of Operative Dentistry, Aichi Gakuin University School of Dentistry, Nagoya 646-8650, Japan; aki-tj@dpc.agu.ac.jp; 6 Minnesota Dental Research Center for Biomaterials and Biomechanics, University of Minnesota, Minneapolis, MN 55455, USA; 7 Evidence-Based Practice Unit, Clinical Sciences Department, Ajman University College of Dentistry, Ajman City P.O. Box 346, United Arab Emirates; 8 Department of Reconstructive Dentistry and Gerodontology, School of Dental Medicine, University of Bern, 3010 Bern, Switzerland

**Keywords:** composite resins, dental bonding, dental ceramics, dental materials, dental porcelain, dental polishing, dental prosthesis, dental prosthesis design, dental veneers, permanent dental restoration, prosthodontics, silicones, surface properties

## Abstract

*Background and Objective:* This study aimed to compare the surface finish of milled leucite-reinforced ceramics polished with ceramic and composite polishing systems based on the manufacturers’ recommendations. *Materials and Methods:* Sixty subtractive computer-aided manufactured (s-CAM) leucite-reinforced glass-ceramic specimens (IPS-Empress-CAD) were assigned into six groups: no polishing, a ceramic polishing kit, and four composite kit groups. The roughness average (Ra) was evaluated in microns using a profilometer, and scanning electron micrographs were obtained for qualitative analysis. A Tukey HSD posthoc test (α = 0.05) was used to determine significant intergroup differences. *Results:* After surface evaluation of the ceramics, the Ra values of the polishing systems ranked OptraFine (0.41 ± 0.26) < Enhance (1.60 ± 0.54) < Shofu (2.14 ± 0.44) < Astropol (4.05 ± 0.72) < DiaComp (5.66 ± 0.62) < No Polishing (5.66 ± 0.74). *Discussion:* Composite polishing systems did not provide as smooth surfaces as the ceramic polishing kit for CAD-CAM leucite-reinforced ceramics. Thus, using ceramic polishing systems, polishing leucite ceramics is recommended, whereas composite polishing systems should not be considered as an alternative for use in minimally invasive dentistry.

## 1. Introduction

The field of dentistry underwent a massive transformation with the advent of computer-aided design and computer-aided manufacturing (CAD-CAM) systems, marking a revolutionary milestone in the late 1980s [1]. Initially, these systems were limited to fabricating inlays. However, their capabilities swiftly expanded to encompass full-coverage crowns and even more intricate restorations, leaving an indelible mark on the dental industry [2]. In the wake of its introduction, the adoption of CAD-CAM technology experienced a surge within clinical and laboratory settings, owing to its reputation for delivering predictable, rapid, and precise outcomes [3,4,5,6]. Dental clinicians and technicians alike were captivated by the remarkable potential of this innovative approach, embracing its ability to streamline workflows and enhance overall treatment efficacy. The versatility of CAD-CAM dentistry has been instrumental in catering to diverse patient needs, offering clinicians an extensive assortment of material options that were previously unimaginable [7,8,9,10]. Indeed, the materials compatible with CAD-CAM dentistry have witnessed a tremendous expansion, providing dental professionals with an extensive palette to craft restorations. Resin composites, known for their life-like aesthetics and improvements in their longevity, have emerged as a popular choice for achieving seamless blends with natural dentition. Glassy ceramics, with their exquisite translucency and ability to replicate natural tooth shades, have garnered substantial recognition for their exceptional aesthetic results. Meanwhile, zirconia, celebrated for its outstanding strength and durability, has revolutionized restorative dentistry by offering an ideal balance between functionality and aesthetics [7,8,9,10]. As CAD-CAM technology advances and evolves, the possibilities for material selection and restoration design have become virtually boundless. This expansion has propelled dentistry into a new era of personalized, patient-centred care, where restorations can be precisely tailored to meet individual requirements. By leveraging CAD-CAM systems, dental professionals can now achieve unrivalled levels of precision, ensuring the optimal fit, form, and function of restorations while reducing chairside time and enhancing patient satisfaction.

Leucite-reinforced glass-ceramic blocks have emerged as a premium material within the realm of subtractive computer-aided manufacturing (s-CAM) restorations, offering a multitude of advantages [11]. This material has gained renown for its remarkable ability to deliver superior aesthetic results, primarily due to its exceptional translucency, which allows for integration with the surrounding dentition [12]. Furthermore, the option to apply stains to the glass structure provides clinicians with the opportunity to meticulously replicate the nuanced shades of natural teeth, thereby further enhancing the overall aesthetic outcome. Regarding mechanical strength, leucite-reinforced ceramics exhibit commendable levels that surpass those of conventional feldspathic porcelain by a factor of two. This remarkable strength renders them a reliable choice for a variety of restorations. However, it is crucial to consider certain limitations when contemplating their use in posterior restorations with extensive spans [13]. While leucite-reinforced ceramics may not possess the necessary robustness for fixed posterior restorations requiring long-span coverage, their physical and mechanical properties make them highly suitable for single-unit prostheses, including laminate veneers, inlays, onlays, and crowns [14].

A detailed examination of leucite-reinforced glass-ceramic materials unveils their unique composition, characterized by the presence of tetragonal leucite crystals, which account for up to 45% of the volume. These crystals are uniformly dispersed within the glass matrix but exhibit an arrangement resembling strings of beads along the grain boundaries. Notably, many of these crystals assume a needle-like morphology and often protrude from the surface, leading to microscopic roughness. Restorations featuring such rough ceramic surfaces can inadvertently give rise to a range of clinical issues, including gingival inflammation, irritation to the tongue and lips, plaque (biofilm) accumulation, and increased wear of opposing teeth [15,16,17]. Addressing these concerns is crucial to ensure optimal patient-reported outcomes measures (PROMs). Conversely, the importance of achieving smooth ceramic surfaces must be considered. Smoothness plays a pivotal role in reducing bacterial adhesion, minimizing plaque retention, and mitigating the propensity for restored teeth to become stained over extended periods of time [18]. Moreover, a smoother ceramic surface can help minimize potential damage to opposing teeth and restorations, particularly considering that reinforced ceramics possess higher hardness compared to common restorative materials such as resin-based composites. While the hardness of reinforced ceramics is comparable to that of enamel, concerns persist regarding the potential long-term impact of a rough ceramic surface on opposing enamel surfaces. Thus, a comprehensive understanding of the intricate interplay between surface characteristics, restoration materials, and their interactions with oral tissues is paramount in achieving long-lasting, functional, and aesthetically pleasing outcomes.

Achieving optimal aesthetic results heavily relies on the meticulous process of polishing [19]. Therefore, it is important to employ a polishing kit that is specifically designed for the type of material used in the restoration. While polishing kits are available in the market tailored for specific materials, the vast array of restorative materials poses a challenge for clinicians, especially those operating in smaller clinics or with solo practices, as maintaining a comprehensive stock of all possible polishing materials becomes impractical. In such cases, if comparable results can be achieved using polishing kits intended for other materials, clinics can expand their range of restorative options while relying on a more limited selection of polishing systems. However, the need for more available information on comparing the surface characteristics of CAD-CAM leucite-reinforced ceramics after polishing with different polishing kits hinders the ability of clinicians to offer restorative materials that could benefit their patients confidently. Novel research is needed to offer guidance to clinicians.

Notably, there is a gap in the existing body of evidence comparing composite and ceramic polishing systems specifically for chair-side CAD-CAM leucite ceramics. Therefore, this novel study evaluates whether composite polishing systems can yield similar finished surfaces for subtractively manufactured (i.e., milled) leucite-reinforced ceramics compared to surfaces achieved with a ceramic polishing system, per the manufacturers’ recommendations. The first null hypothesis posits that the four composite polishing kits would generate comparable surface roughness to that achieved with the ceramic polishing kit on CAD-CAM leucite-reinforced ceramics. The second null hypothesis assumes that no significant differences in surface roughness would be observed among the four composite kits utilized in this study.

A comprehensive investigation was undertaken to address these research objectives and test the hypotheses, considering the specific context of CAD-CAM leucite-reinforced ceramics. This study encompassed an examination of the surface roughness obtained from various composite polishing systems and a ceramic polishing system, aiming to shed light on the efficacy of potential substitutions and their impact on the final surface characteristics of the restorative material. By delving into this unexplored area, we novelly sought to provide valuable insights into the selection of polishing systems, enabling dental clinicians to make informed decisions when choosing restorative materials and ensuring the delivery of high-quality care to their patients.

## 2. Materials and Methods

### 2.1. Specimen Preparation

Sixty flat samples (2 mm thick) were fabricated from s-CAM leucite-reinforced glass-ceramic blocks (IPS Empress CAD, Ivoclar Vivadent, Schaan, Liechtenstein) for surface evaluation. The samples were divided into six groups: (1) No polishing (NP); (2) Polished using the OptraFine ceramic polishing kit (Ivoclar Vivadent, Schaan, Liechtenstein) (OP); (3) Polished using the Astropol composite polishing kit (Ivoclar Vivadent, Schaan, Liechtenstein) (AP); (4) Polished using the DiaComp composite polishing kit (Brasseler USA, Savannah, GA, USA) (DP); (5) Polished using the Enhance composite polishing kit (Dentsply Sirona, Charlotte, NC, USA) (EP); (6) Polished using the Shofu composite polishing kit (Shofu, Kyoto, Japan) (SP). Each polishing treatment was performed based on the manufacturers’ instructions. The materials used to polish the ceramic and composite specimens are described in Table 1.

### 2.2. Profilometer Analysis 

Profilometer analysis was conducted with a 3D non-contact optical profilometer (Contour GT-K 3D Optical Microscope, Bruker, Billerica, MA, USA) after ultrasonic cleaning for 1 min. Ten samples were scanned for each group. Samples were scanned and measured by a vertical scan using a 5× magnification lens with a 1 mm × 1 mm field of view. Ra (roughness average), Rp (maximum peak height), and Rv (maximum valley depth) were used to quantify the surface topography of each scan.

### 2.3. Scanning Electron Microscopy (SEM) Observations

Samples were ultrasonically cleaned for 1 min to remove contamination introduced by handling and then mounted in a holder stub for gold sputtering. Micrographs were obtained with a scanning electron microscope (Jeol JSM-6610LV, Jeol, Tokyo, Japan) at 500× using an accelerating voltage of 15 kV.

### 2.4. Statistical Analysis 

The normality and homogeneity of variance of the data were confirmed, and a one-way ANOVA test was used to determine whether there was a significant difference in the surface roughness between polishing systems. A Tukey HSD posthoc test (α = 0.05) was used to determine which polishing kits showed significant intergroup differences. The analyses were performed with the Statistical Package for the Social Sciences (IBM SPSS version 26.0; SPSS Inc. Chicago, IL, USA). Values are shown as the mean and standard deviation.

## 3. Results

**Figure 1:**
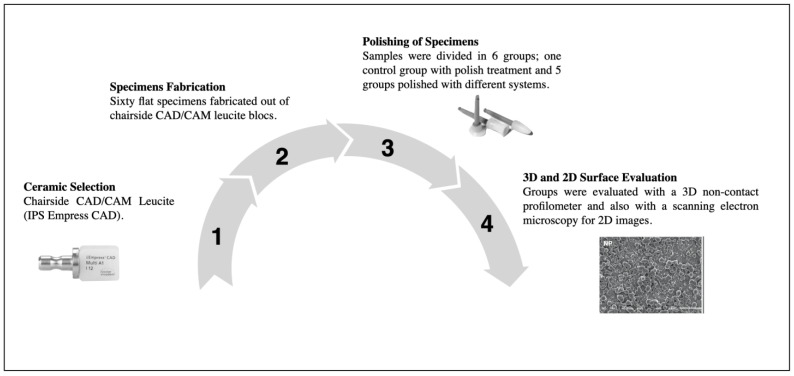
Description of the workflow: 1. Selection of the chairside CAD-CAM leucite ceramic (IPS Empress CAD, Ivoclar Vivadent); 2. Specimens’ fabrication: sixty flat specimens with a 2 mm thickness were fabricated out of the CAD-CAM blocs; 3. Specimens were divided into six groups and polished following manufacturers’ recommendations; 4. Specimens were evaluated with an optical profilometer and scanning electron microscope for a both 3D and 2D comparison, respectively.

A description of the workflow for the evaluation of ceramic and composite resin polishing systems on the surface roughness of milled leucite-reinforced ceramics is provided in Figure 1.

### 3.1. Surface Roughness

Results for the effectiveness of ceramic and composite surface-polishing systems for CAD-CAM leucite-reinforced glass-ceramic restorations are shown in Table 2. Values obtained from optical profilometry for Ra, Rp, and Rv are shown.

The rank order for roughness average values (Ra) was OP < EP < SP < AP < DP < NP. All groups’ Ra values were statistically significantly different from each other except EP and SP. Statistically significant differences were observed in roughness average (Ra), maximum peak height (Rp), and maximum valley depth (Rv) values between OP and others. OP showed statistically significant lower values in Ra, Rp, and Rv. No statistically significant differences existed between composite kits, and the NP group was seen for Rp or Rv. The black areas in the micrograph are the edges of the scan.

### 3.2. Profilometer Observations 

**Figure 2:**
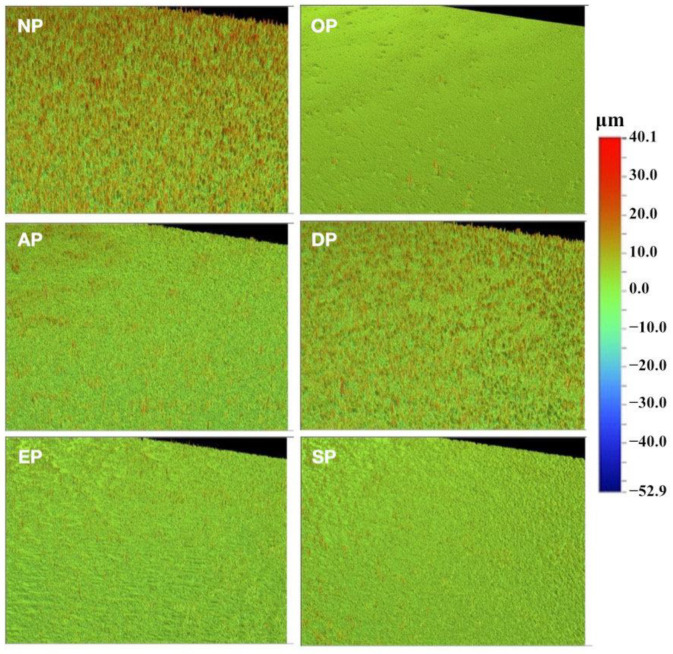
Profilometer micrographs of CAD-CAM leucite-reinforced ceramic surface without and with polishing treatment. NP, no polishing; OP, OptraFine ceramic; AP, Astropol composite; DP, DiaComp composite; EP, Enhance composite; SP, Shofu composite polishing kit. Scale bar for comparison is shown in μm for all micrographs.

The profilometer observations of prepared leucite-reinforced ceramic samples are shown in Figure 2. Profilometer micrographs showed three general groups of surfaces. A much smoother surface was observed in OP than in the others, while AP, EP, and SP looked very similar, and DP showed little difference from NP.

### 3.3. Scanning Electron Microscopy Observations

**Figure 3:**
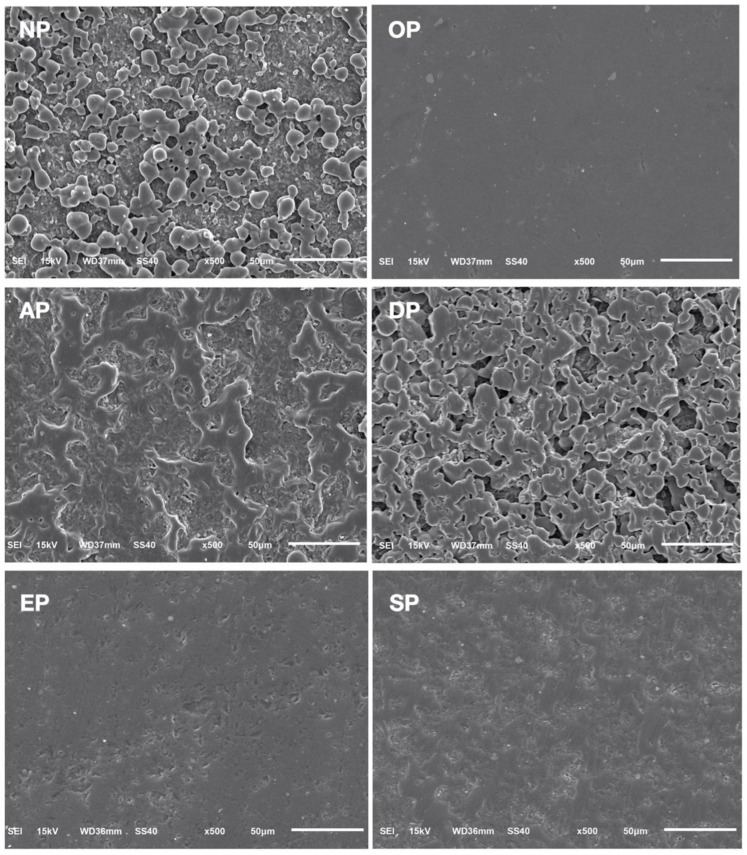
Scanning electron micrographs of CAD-CAM leucite-reinforced ceramic surface without and with polishing treatment. NP, no polishing; OP, OptraFine ceramic; AP, Astropol composite; DP, DiaComp composite; EP, Enhance composite; SP, Shofu composite polishing kit. The scale bar is 50 μm for all micrographs obtained at 500×.

SEM observations of prepared leucite-reinforced ceramic samples are shown in Figure 3. The polished surfaces appear to fall into, again, three groups. OP appeared much smoother than the others, while EP and SP were similarly devoid of notable topographic features. AP and DP closely resembled the no-polishing group, NP, compared to all other polishing groups.

## 4. Discussion

Surface roughness analysis is a crucial aspect of evaluating the effects of various polishing techniques, with Ra being a widely accepted measurement parameter [18]. Ra represents the average absolute deviation of the surface from its mean over the measured line length, providing a quantitative assessment of surface roughness [18,19]. Additionally, Rp and Rv offer further insights into the topographic changes that occur on the surface following polishing. Profilometer and scanning electron microscopy (SEM) observations were employed to gain a comprehensive understanding of the ceramic surface, as these techniques are commonly utilized for the two-dimensional image evaluation of polished surfaces [20]. While profilometer measurements provide quantitative data, SEM micrographs offer qualitative information on surface morphology, complementing the profilometer analysis [21]. In this study, the obtained micrographs exhibited high consistency with the quantitative measurements, reinforcing the reliability of the applied procedures. Therefore, based on previous investigations, Ra was selected as the primary outcome measure [18,19,20,21].

The results pertaining to Ra demonstrated that the ceramic polishing kit, which involved the utilization of diamond paste and OP, yielded a significantly lower roughness average (0.41 ± 0.26 μm) compared to the four composite polishing kits tested. Among the composite kits, EP (1.60 ± 0.54 μm) and SP (2.14 ± 0.44 μm) exhibited similar Ra values, both of which were significantly lower than those observed in the remaining groups. Notably, AP displayed a notably higher Ra value (4.05 ± 0.72 μm), although it was still significantly lower than the control group that underwent no polishing treatment, NP (5.66 ± 0.62 μm). Furthermore, the Ra value obtained for the polishing treatment DP did not significantly differ from the control group (5.66 ± 0.62 µm). These findings are visually depicted in the micrographs, where distinguishing DP from NP becomes challenging, as does differentiating between EP and SP. Conversely, AP exhibited closer similarities to EP and SP in the profilometer micrographs, while displaying more resemblances to DP and NP in the SEM micrographs. On the other hand, OP exhibited a smoother surface compared to all the other groups in both observations.

These results highlight the distinct impact of various polishing kits on the surface roughness of CAD-CAM leucite-reinforced ceramics. The superior performance of the ceramic polishing kit, in conjunction with the diamond paste and OP, is evident in the significantly lower Ra values obtained. Meanwhile, the composite polishing kits displayed varying degrees of surface roughness, with EP and SP producing comparatively smoother surfaces. The higher Ra values observed for AP suggest the potential limitations of using composite polishing kits for achieving optimal surface smoothness. Notably, the control group without any polishing treatment, NP, exhibited the highest Ra value, emphasizing the importance of proper polishing techniques in attaining desired surface characteristics. The micrographs visually corroborate the quantitative measurements, comprehensively representing the surface topography. These findings contribute to understanding the polishing options available for CAD-CAM leucite-reinforced ceramics and their implications for achieving desirable surface roughness.

The first null hypothesis of the study, which stated that “the four composite polishing kits would create the same surface roughness as the ceramic polishing kit on CAD-CAM leucite-reinforced ceramics”, was rejected based on the obtained results. Previous investigations have reported that the Ra values of normal enamel surfaces typically range from 0.45 to 0.65 μm [22]. Interestingly, unlike the composite polishing kits, the ceramic polishing system utilized in this study demonstrated the ability to achieve a surface roughness comparable to intact enamel. Furthermore, a prior study indicated that the Ra values of leucite-reinforced ceramic surfaces after glazing ranged from 0.29 to 0.41 μm [23]. Consequently, if the glazed surface is removed during occlusal adjustment, nearly identical surface roughness can be attained using the ceramic polishing system.

The findings supported the rejection of the second null hypothesis, which posited that “there would be no differences in surface roughness among the four composite kits” employed in this study. Although the Ra results suggest that EP and SP approaches were approaching an acceptable level of surface roughness, a comprehensive analysis of the Rp and Rv results suggests that such a conclusion may be premature. Specifically, only OP exhibited a significant difference in Rv, as depressions on the surface were not filled or removed during the polishing process. Consequently, a reduction in Rv is expected only when the entire surface is significantly diminished. Furthermore, OP led to a highly significant reduction in Rp, with a value of 23.44 ± 8.60 µm, in comparison to the unpolished surface’s Rp value of 39.21 ± 3.72 µm. Conversely, the composite polishing groups did not exhibit significant differences from the unpolished group in terms of Rp. These findings indicate that the composite polishing treatments failed to reduce the length of protrusions on the surface. Since damage to opposing surfaces is predominantly attributed to contact with protrusion excursions, it can be inferred that the composite polishing systems did not effectively create an acceptable surface for clinical applications.

These results shed light on the implications of utilizing different polishing kits for CAD-CAM leucite-reinforced ceramics. The rejection of the first null hypothesis underscores the superior performance of the ceramic polishing system, which successfully achieved surface roughness akin to that of intact enamel. In contrast, the composite polishing kits failed to achieve comparable results, emphasizing the limitations of these systems in attaining optimal surface characteristics. Similarly, the rejection of the second null hypothesis highlights the differences in surface roughness among the four composite kits tested. Although the Ra values for EP and SP appeared promising, a more comprehensive analysis considering Rp and Rv indicated that these kits might not yield satisfactory surface smoothness. On the other hand, OP demonstrated significant improvements in both Rp and Rv, suggesting its potential as a viable alternative to the ceramic polishing system. These findings have important implications for clinical practice, as they inform dentists about the most effective polishing options for CAD-CAM leucite-reinforced ceramics, ultimately enhancing the quality of restorative procedures and patient outcomes.

Studies focusing on evaluating composite and ceramic polishing systems are limited in the existing literature, highlighting the need for further investigation in this area. However, a recent study comparing resin composite and ceramic polishing systems for chairside CAD-CAM lithium disilicate restorations concluded that the ceramic polishing system exhibited higher effectiveness compared to the composite polishing system [9]. Similarly, another study comparing ceramic and composite polishing systems for CAD-CAM polymer-infiltrated ceramics found that while composite systems improved surface roughness, ceramic systems were more reliable and provided smoother surfaces [19]. Our findings align with these studies, as the composite polishing systems demonstrated improvements in surface smoothness compared to the control groups without polishing. In contrast, the ceramic polishing system yielded superior results. Our studies may also be useful to inform of the finishing and polishing of custom-made abutments for implants; the surface roughness is critical to both reduce plaque formation but also encourages soft tissue healing [24].

The primary distinction between the ceramic polishing kit employed in our study and the composite polishing systems was incorporating a diamond polishing paste for the final polishing stage in the ceramic kit. In contrast, the composite polishing kit utilized a silicone polisher for the final polishing step. Considering the evident differences observed among the composite polishing kits, it is possible that the systems that exhibited better performance could potentially achieve entirely satisfactory results if a final stage of polishing with diamond paste were introduced. Therefore, further research should investigate the feasibility of developing a universal polishing kit based on one of the composite surface-polishing systems, considering the potential benefits it may offer in clinical practice.

Future studies should include other brands of ceramic polishing systems to evaluate their differences comprehensively. Moreover, our study did not assess the surface smoothness of the ceramics with a glazing application, which presents an exciting avenue for future research. Additionally, evaluating other chairside CAD-CAM leucite ceramic brands using the same methods and comparing them under standardized experimental conditions (e.g., rotary instrument speed, water supply, abrasion time, and abrasion pressure) and following the manufacturers’ recommendations would provide valuable insights. Furthermore, conducting in silico studies would offer added value by corroborating the findings from our and other in vitro studies focused on the surface treatment of dental materials. Therefore, incorporating in silico studies is recommended to validate the results obtained from in vitro studies and enhance our understanding of surface treatment techniques for indirect permanent dental restorations.

## 5. Conclusions

In conclusion, using both ceramic and composite polishing systems, this comparative in vitro study assessed the surface smoothness of CAD-CAM leucite-reinforced glass-ceramic samples. The results indicated that the ceramic polishing system achieved a surface smoothness similar to normal enamel when applied according to the manufacturers’ recommendations. However, none of the four composite polishing systems tested provided comparable surface smoothness. Moreover, the study findings clearly demonstrated that all the composite polishing systems resulted in significantly rougher surfaces compared to the ceramic polishing system for leucite-reinforced ceramics. This highlights the limitations of using composite polishing systems as an alternative to ceramic polishers for glass-based restorations. Therefore, it is recommended to strictly adhere to ceramic polishing systems when working with leucite-based restorative materials to achieve optimal surface smoothness.

Future studies should consider expanding the assessment of surface smoothness to include the evaluation of ceramics with and without glazing. This would provide valuable insights into the impact of a glazing application on the surface characteristics of leucite ceramics and help inform the selection and optimization of ceramic polishing systems. Additionally, including various brands of ceramic polishing systems in future studies would enable a comprehensive evaluation of their differences and facilitate the identification of the most effective polishing protocols for leucite-based restorations. By advancing our knowledge in this area, dental professionals can enhance their clinical practices and deliver high-quality restorative outcomes to their patients.

## Figures and Tables

**Table 1:** Description of polishing systems used in this study.

Group	Polishing System	Manufacturer	Components	Manufacturer Instructions
*Group 1 (NP)*	No Polishing	None	No Polishing	None
*Group 2 (OP)*	OptraFine Ceramic Polishing Kit	Ivoclar Vivadent, Schaan, Liechtenstein	- Light blue diamond finishers (F) in flame, cup, and disc shapes. - Dark blue diamond polishers (P) in flame, cup, and disc shapes. - Nylon brushes for high-gloss polishing (HP), suitable for use in conjunction with the paste. - Diamond polishing paste for high-gloss polishing (HP).	Step 1: Pre polishing using light blue. Step 2: Polishing using dark blue. Step 3: High-gloss polishing with brushes and paste. Speed: 1000 up to max 1500 rpm for diamond finishers (F) and polishers (P) with copious water spray (>50 mL/min). 7000 up to max 1000 rpm for brushes (HP) and polishing paste without water spray.
*Group 3 (AP)*	Astropol Composite Polishing	Ivoclar Vivadent, Schaan, Liechtenstein	Astropol F (grey), P (green), and HP (pink) shapes: - Small flame, large flame, cup, and disc.	Astropol F (grey) for removal of excess composite material. Astropol P (green) for polishing. Astropol HP (pink) for final high-gloss polishing. Speed: 7000 to 10,000 rpm. Only used with copious water spray (>50 mL/min). Use without polishing paste.
*Group 4 (DP)*	DiaComp Composite Polishing	Brasseler USA, Savannah, GA, USA	Medium (green) and fine (grey) grits for: - Points for the occlusal surface. - Knife edge for proximal, buccal, and lingual. - Cup for following the contour of the tooth and restoration.	Green medium grit is used to remove scratches and stain shine surfaces. Gray fine grit leaves a high shine finish. Speed: 5000 to 6000 rpm. May be used dry with a feather touch or wet.
*Group 5 (EP)*	Enhance Finishing System for Composite Polishing	Dentsply Sirona, Charlotte, NC. USA.	Finishing (white) cups, finishing (white) discs and finishing (white) points.	Use cups, discs, and points with light intermittent pressure. Speed: 10,000–15,000 rpm. Polishers are designed for use without water.
*Group 6 (SP)*	CompoSite Polishing Kit for Composite Polishing	Shofu Inc, Kyoto, Japan	Dura-green stones, dura-white stones, and composite polishers: bullet, knife, and cup shapes.	Dura-green and dura-white for finishing and composite polishers for pre-polishing and polishing. Speed (all in rpm): Dura-green stones 5000–20,000. Dura-white stones 5000–20,000. Composite polishers 10,000–12,000.

Abbreviations: NP, no polishing; OP, OptraFine ceramic; AP, Astropol composite; DP, DiaComp composite; EP, Enhance composite; SP, Shofu composite polishing kit.

**Table 2:** Effectiveness of ceramic and composite surface-polishing systems for CAD-CAM leucite-reinforced ceramic restorations.

Polishing Systems		Surface Evaluation (μm)		
		Ra Mean (SD)	Rp Mean (SD)	Rv Mean (SD)
*NP*	*No kit*	5.66 (0.74) ^a^	39.21 (3.72) ^a^	−50.83 (4.56) ^a^
*OP*	*Ceramic kit*	0.41(0.26) ^b^	23.44 (8.60) ^b^	−41.56 (12.92) ^b^
*AP*	*Composite kit*	4.05 (0.72) ^c^	40.03 (2.29) ^a^	−55.18 (5.12) ^a^
*DP*	*Composite kit*	5.66 (0.62) ^a^	41.41 (4.10) ^a^	−54.22 (5.51) ^a^
*EP*	*Composite kit*	1.60 (0.54) ^d^	35.53 (6.30) ^a^	−57.11 (6.50) ^a^
*SP*	*Composite kit*	2.14 (0.44) ^d^	38.28 (6.72) ^a^	−57.67 (6.25) ^a^

Ra, roughness average; Rp, maximum peak height; Rv, maximum valley depth; SD, standard deviation; NP, no polishing; OP; OptraFine including diamond paste; AP, Astropol; DP, DiaComp; EP, Enhance; SP, Shofu. Different superscript letters denote statistically significant differences (*p* < 0.05) within each column for each surface topography quantification.

## Data Availability

Not applicable.
